# Round the Hospitals

**Published:** 1921-07-16

**Authors:** 


### ROUND THE HOSPITALS.

It is welcome news that, as will be seen from the Minister of Health's reply to the deputation of nurses' organisations on the Registration Act, the Scottish Board of Health has come into line on the question of admitting fever nurses to the General Register, and the Secretary for Scotland has now agreed to withdraw the proposal that Scottish fever nurses should be admitted to the general part of the Register. Thus an important obstacle to complete reciprocity between the two countries has been removed, and Sir Alfred Mond said he anticipated no difficulty in adjusting the minor points still outstanding. With his promise to sign the Rules at once, subject to the reciprocity rule being deferred, and his undertaking to use his influence to secure uniformity of standard, the registration of nurses in England and Wales is now in fair train to be pushed with real vigour. A meeting of the General Nursing Council is being held as we go to press, when, doubtless, further information will be made public. We congratulate the English and Scottish Councils and all concerned on this happy conclusion to what at one time seemed a very awkward *impasse*.

**ROUND THE HOSPITALS—(*continued*).**

From information which reaches us as we go to press we regret to learn that Miss Cox-Davies, whose absence from the annual meeting on Saturday of the Association of Hospital Matrons was deplored by all, is very ill, and her condition shows no change. The sympathy of nurses, and particularly of members of the College of Nursing, will go out to her in full measure, with earnest wishes for her speedy recovery.

The historic garden-party, held at St. James's Palace on July 7 to celebrate the Jubilee of St. Thomas's Hospital on its present site, was graced by the presence of the Queen, Princess Christian, Lady Patricia Ramsay, the Princesses Helena Victoria and Marie Louise, and lacked nothing which could contribute to its success. Choice entertainments of music and dancing, among which a set of glees sung by members of the nursing staff was much appreciated, were given on the terrace. Miss Phyllis Broughton presided at a wheel of fortune, at which the Queen won a cake of soap, and Chester, the celebrated chef of Sir W. Orpen's picture, had the honour of selling Her Majesty some of the post-cards with which he secured a considerable sum for the hospital. A very distinguished company gathered under the trees for tea, and among them many notable figures in the nursing world.

Statements have been circulated in the lay Press relating to "nurses' long hours at Lambeth Infirmary," which are stated as fifty-seven hours' work a week for staff nurses and probationers on day duty, and seventy-two for night nurses. No allusion is made to the fact that day nurses have one whole day a week off duty and night nurses twenty-four hours on completion of each week's duty. The problem of further reducing the hours on duty is complicated by housing difficulties, but when the proposed amalgamation with the institution across the road takes place there will be accommodation for more nurses and conditions will be improved. The twelve hours' night duty, however, is so serious a strain on the probationers that we hope to hear means have been found to shorten it before the other alterations take place.

An interesting appointment has been made by the committee of the Royal Halifax Infirmary. They have appointed a sister-tutor to undertake the instruction of the nurses in the preliminary school, and she will also help to coach the other nurses. She will take up her duties in October next. The preliminary training-school is a new departure which is found necessary in order to carry out the Draft Syllabus. Another change in this training-school is the extension of the training period to four years, during which time midwifery training will be given. Halifax Royal Infirmary is thus showing itself thoroughly progressive, and its action should add to its already high reputation as a major training school.

Edinburgh nurses are to be congratulated on the immediate and convincing success of the College Club at Drumsheugh Gardens. Financially it has beaten all expectations. At the annual meeting of the Club, held on June 29, Miss Gill, R.R.C., presided, and the report was read by Lady Wallace. There are now 525 members, of whom 479 are trained nurses and the rest probationers, while applications are constantly coming in. The balance sheet shows a sum of nearly £2,000 in hand. The membership fee of 10s. 6d. suffices to meet the whole of the expenses and half the rent, from which it can surely be deduced that the managers understand what they are about.

District nurses' work is to a large extent hidden work, and their names are seldom heard outside a very small sphere. It is all the more commendable that Dr. Bullough, County Medical Officer of Health, should have called attention at the annual meeting of the Sussex County Nursing Association to the good work accomplished by "Sister Alice" and her colleagues, Sister Rose and Sister Ross. In moving a special vote of thanks to them, he alluded to "Sister Alice" in particular as a considerable asset to the country, who not only trains nurses, but inspires them with the true spirit of nursing. In all 513 nurses have passed under her care, and it is a tribute to her methods that the difficulty of getting suitable women for training is now vanishing; a good number of applications have come in, and the two homes are full of nurses in training.

The people of Croydon, with the co-operation of Dr. Veitch Clark, M.O.H., are making plans for establishing a complete nursing service for the sick and infirm of all classes of the community. It is estimated that a superintendent and six nurses will be required to begin with, and, pending the acquirement of a central home, the nurses will be housed in their own rooms in different parts of the town. The service, we rejoice to hear, is to be to a very large extent self-supporting. There will be a graduated scale of fees and provident schemes for including all within the benefits. The efficiency of the service will be guaranteed by affiliation to the Jubilee Institute. An initial sum of £3,000 is asked for to float the service, and we trust will shortly be obtained.

